# An Intrahepatic Fluorodeoxyglucose (FDG)-PET/CT False-Positive Tumor Secondary to Foreign Body Granuloma Masquerading as Colon Cancer Liver Metastasis: A Case Report

**DOI:** 10.7759/cureus.52657

**Published:** 2024-01-21

**Authors:** Takehiko Hanaki, Soichiro Honjo, Mikiya Kishino, Yuki Murakami, Manabu Yamamoto, Tokuyasu Naruo, Teruhisa Sakamoto, Toshimichi Hasegawa, Yoshiyuki Fujiwara

**Affiliations:** 1 Department of Gastrointestinal and Pediatric Surgery, Tottori University Faculty of Medicine, Yonago, JPN; 2 Department of Surgery, Matsue City Hospital, Matsue, JPN

**Keywords:** hepatectomy, schloffer’s tumor, positron emission tomography/computed tomography, foreign-body granuloma, colon cancer liver metastasis

## Abstract

A suture placed next to a dissected liver section during the initial hepatectomy may become an unlikely intrahepatic foreign body granuloma. In this report, we describe a case where a silk suture in the liver section plane placed during initial hepatectomy for synchronous colon cancer metastasis became an intrahepatic foreign body granuloma that exhibited fluorodeoxyglucose (FDG) accumulation on positron emission tomography/computed tomography (PET/CT). The granuloma was resected as the second metachronous liver metastatic lesion. A 73-year-old female was referred for a planned second hepatectomy. She had undergone colectomy and hepatectomy for advanced cancer of the ascending colon and synchronous liver metastasis approximately two years ago. However, two possible liver metastases with FDG accumulation were identified in hepatic segments IV and V after one year and nine months after the initial resection. A second hepatectomy was planned after administering systemic chemotherapy. She underwent a left lobectomy with a middle hepatic vein and partial segment V hepatectomy six months after liver lesion identification. The segment IV lesion was histologically proven to be a liver metastasis adenocarcinoma. The segment V lesion revealed a silk thread on the residual liver side at the initial hepatectomy, which was histologically diagnosed as a foreign body granuloma. The possibility of intrahepatic foreign body granuloma development should be considered in subsequent follow-ups in cases where sutures were applied to the dissected residual liver plane during the initial hepatectomy. Additionally, a thorough second hepatectomy should be considered if recurrence is suspected.

## Introduction

Foreign body granulomas are postoperative granulomas that may develop because of surgical materials [1,2]. Schloffer reported a mass in a patient’s wound after performing an inguinal hernia surgery in 1909, which developed due to the ligature suture [3]. Such a mass is thus occasionally called Schloffer’s tumor.

Traction by placing transparenchymal sutures over the resected and remaining liver is a widely used pretreatment technique in liver surgery that may help in preventing bleeding during liver dissection and postoperative bile leakage [4,5]. Although pre-suturing procedures for hepatic dissection are widely performed, complications associated with this procedure have been reported in some cases.

Here, we report a case in which a second hepatectomy was performed simultaneously for a metachronous metastatic liver lesion and an iatrogenic liver lesion, which was a Schloffer’s tumor that showed fluorodeoxyglucose (FDG) accumulation.

## Case presentation

**Figure 1:**
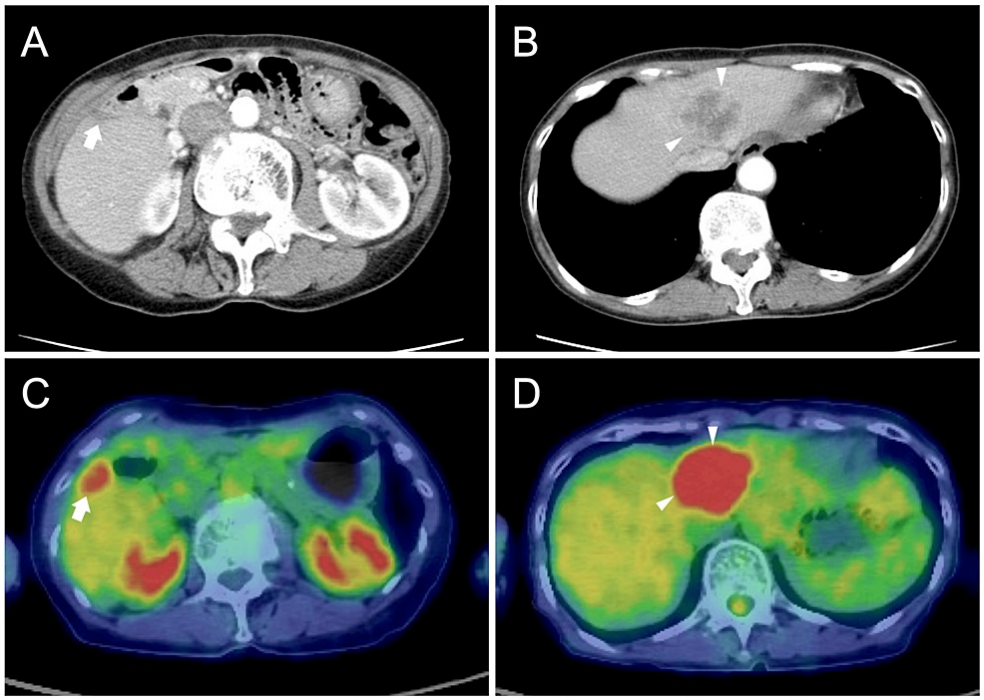
Diagnostic images during metachronous liver recurrence diagnosis. Contrast-enhanced CT shows a hypovascular tumor (arrow) in segment V of the liver surface (A) and a hypovascular tumor (arrowheads) with peripheral enhancement in segment IV (B). PET/CT shows the maximum standard uptake value (SUVmax) at the segment V lesion was 3.87 (C, arrow) and the SUVmax at the segment IV lesion was 12.5 (D, arrowheads).

A 73-year-old female was hospitalized for a second hepatectomy for metachronous metastatic cancer. Approximately two years before presentation, she had undergone open right hemicolectomy and partial hepatectomy of the liver surface in segment V in another hospital for an ascending colon advanced carcinoma and its synchronous liver metastasis. After the initial colectomy and hepatectomy, tegafur-uracil-leucovorin therapy was administered as adjuvant chemotherapy for six months. Lesions were detected using computed tomography (CT) in liver segments V and IV (Figure 1A, 1B) 21 months after the initial surgery.

**Figure 2:**
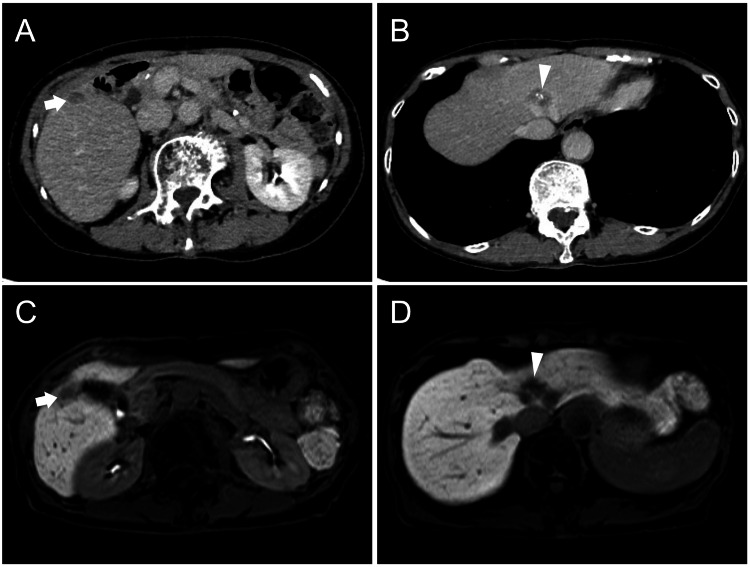
Diagnostic images before the second hepatectomy. Contrast-enhanced CT (A, B) and gadolinium ethoxybenzyl diethylenetriamine pentaacetic acid-enhanced MRI (C, D): The lesion in segment V (arrow) is oval-shaped and slightly enlarged (A); however, the lesion in segment IV (arrowhead) has markedly shrunk in response to chemotherapy (B). The lesion in segment V appears to be within the liver parenchyma (C). The lesion in segment IV is in contact with the root of the middle hepatic vein and the left hepatic vein (D).

Both lesions showed FDG accumulation on PET/CT (Figure 1C, 1D). She was diagnosed with metachronous liver metastatic recurrence, and four cycles of systemic chemotherapy (capecitabine and oxaliplatin) were administered. Chemotherapy reduced the segment IV lesion and slightly enlarged the segment V lesion (Figure 2).

There was no change in tumor marker levels at the initial surgery, recurrence, and second surgery. We decided to perform hepatectomy for both lesions because no other intrahepatic and extrahepatic lesions were detected.

**Figure 3:**
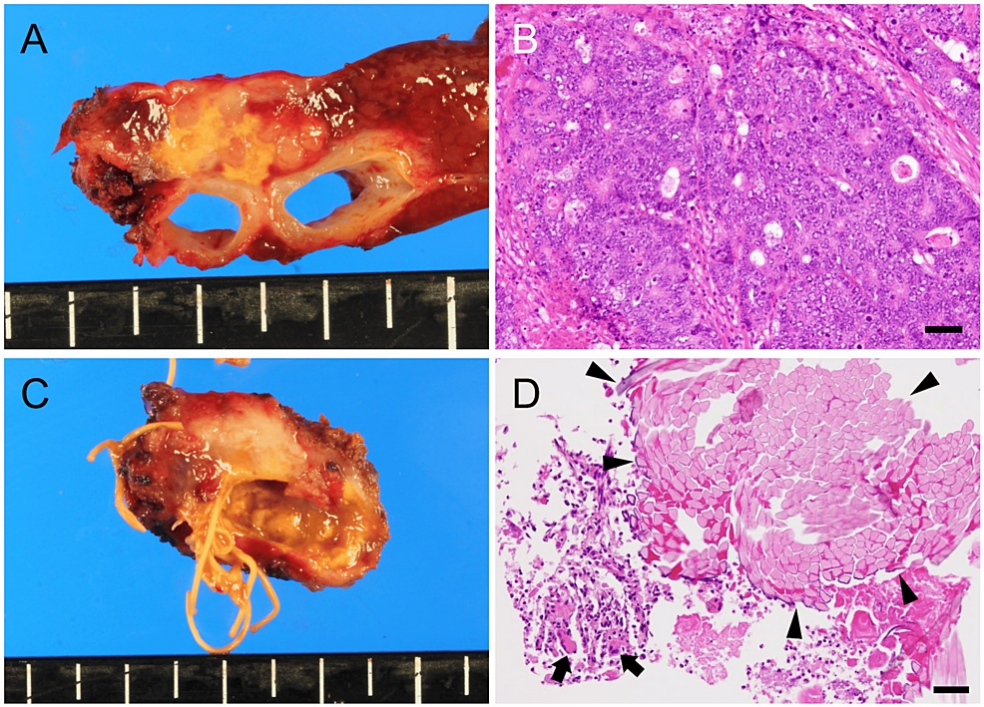
Resected specimens and pathological examinations. Macroscopic tumor observation located in segment IV (A). The tumor borders the left hepatic and middle hepatic veins. Pathologically, the diagnosis was a moderately to poorly differentiated adenocarcinoma liver metastasis (B). The gross observation of segment V lesion’s divided surface shows silken threads originating from the initial hepatectomy (C). Histologically, it also reveals multinucleated giant cells (arrows) and silk thread fragments (arrowheads) with inflammatory cell infiltration (D). Bars, 50 μm (B, D).

Left hepatic lobectomy with middle hepatic vein reconstruction using a left portal vein branch autograft and partial segment V resection were performed six months after the recurrence was diagnosed, because the segment IV lesion was in contact with the middle hepatic vein root and left hepatic vein. Surgery was completed without residual tumor. The surgery lasted for 295 minutes, with an intraoperative blood loss of 195 mL. Pathologically, the segment IV lesion was preoperatively diagnosed as liver metastasis of adenocarcinoma (Figure 3A, 3B).

The segment V lesion was diagnosed as a foreign body granuloma caused by a silk thread core placed in an isolated liver section during the initial hepatectomy (Figure 3C, 3D). The patient was discharged on the ninth postoperative day and thrived for 40 months with no evidence of recurrence after the second hepatectomy.

## Discussion

Foreign body granulomas develop because of an inflammatory reaction against a foreign body left in the body after surgery, commonly referred to as Schloffer’s tumor [2,3]. Silk threads are a commonly used surgical material with non-absorbable properties to the organism. The incidence rate of foreign body granuloma caused by silk threads embedded in the abdominal wall is reportedly 0.61% [6], suggesting that foreign body granulomas are rarely encountered. There are several reports of foreign body granulomas against sutures or staples left during surgery in the body wall or body cavity [1,7,8]. PET/CT can also generate false positive results, making it challenging to distinguish foreign body granulomas from true recurrent malignancy [9,10]. Another reason for difficulty in differentiating foreign body granulomas from recurrent malignancy is their rapid growth rate [10]. A mass near a section plane of the liver is often initially considered a possible liver metastatic recurrence associated with tumor remnant after resecting metastatic lesions in the liver or after resecting primary liver cancer.

 In our case, although one of the two liver lesions was a foreign body granuloma and the other was a metastatic liver recurrence, differentiating them preoperatively was challenging. There have been reports of intrahepatic stone development due to sutures [11-13]; however, in this case, the intrahepatic mass was formed due to silk sutures that were placed to compress the liver parenchyma. Our institution has stopped adding compression sutures to the residual liver for remnant liver parenchyma as a pre-hepatectomy treatment to prevent bleeding and bile leakage based on this experience. Without suture placement and its traction, we were able to perform hepatectomy without any issues. Use of absorbable threads rather than non-absorbable silk threads while adding sutures to the residual liver may also help prevent foreign body granuloma development and decrease the risk of surgical site infection [5]. It is crucial to be mindful of the possibility of non-absorbable thread-induced mass formation after hepatectomy performed at other institutions. Although it is unlikely that a foreign body granuloma will result from a suture placed in an isolated liver section, it was observed in this case. This is a rare case of a foreign body granuloma that appeared to be an intrahepatic lesion on preoperative imaging. However, many cases of foreign body granulomas can be identified as lesions on the liver surface, body cavity, or body wall. In patients who have undergone hepatectomy, intrahepatic lesions located close to the liver surface may be caused by intrahepatic compression suture placement during the initial hepatectomy.

## Conclusions

We described a case in which a suture placed in the liver at the initial hepatectomy resulted in an intrahepatic foreign body granuloma with FDG accumulation. The possibility of intrahepatic foreign body granuloma appearance should be considered in subsequent follow-ups where sutures were applied to the dissected residual liver plane at the time of initial hepatectomy. Additionally, a thorough second hepatectomy should be considered in case of any suspected recurrence.

## References

[1] Pantiora EV, Kontis EA, Michalaki V, Primetis E, Vezakis A, Polydorou A, Fragulidis GP (2016). Granuloma mimicking local recurrence on pet/ct after liver resection of colorectal liver metastasis: a case report. Cureus.

[2] Takano Y, Haruki K, Tsukihara S (2021). Suture granuloma with hydronephrosis caused by ileostomy closure after rectal cancer surgery: a case report. Surg Case Rep.

[3] Schloffer H (1909). About chronic inflammatory abdominal wall tumors and hernia operations [Article in German]. Arch Klin Chir.

[4] Wilson H, Gillespie CE (1949). Partial hepatectomy with intrahepatic cholangiojejunostomy : a useful addition to technical methods for the management of common duct stricture. Ann Surg.

[5] Dou L, Liang HF, Yang HY, Ji R, Chen YF, Chen XP (2019). Clinical value of trans-parenchymal compressing suture to decrease the cutting surface related complications after non-anatomical liver resection. Curr Med Sci.

[6] Nagar H Stitch granulomas following inguinal herniotomy: a 10-year review. J Pediatr Surg.

[7] Nakasuka T, Fujimoto N, Hara N (2015). Foreign body granuloma mimicking recurrence of malignant pleural mesothelioma. Respir Med Case Rep.

[8] Kim SW, Shin HC, Kim IY, Baek MJ, Cho HD (2009). Foreign body granulomas simulating recurrent tumors in patients following colorectal surgery for carcinoma: a report of two cases. Korean J Radiol.

[9] Yüksel M, Akgül AG, Evman S, Batirel HF (2007). Suture and stapler granulomas: a word of caution. Eur J Cardiothorac Surg.

[10] Matsuura S, Sasaki K, Kawasaki H, Abe H, Nagai H, Yoshimi F (2016). Silk suture granuloma with false-positive findings on PET/CT accompanied by peritoneal metastasis after colon cancer surgery. Int J Surg Case Rep.

[11] Kinoshita H, Sajima S, Hashino K (2000). A case of intrahepatic gallstone formation around nylon suture for hepatectomy. Kurume Med J.

[12] Katsuki T, Tanikawa H, Sato S, Nakagawa N (1986). Cholesterol gallstone of the bile ducts with a silk suture in its center--report of two cases [Article in Japanese]. Nihon Shokakibyo Gakkai Zasshi.

[13] Li Q, Tao L, Wu X, Mou L, Sun X, Zhou J (2016). Bile duct stone formation around a Prolene suture after cholangioenterostomy. Pak J Med Sci.

